# ACDF versus corpectomy in octogenarians with cervical epidural abscess: early complications and outcomes with 2 years of follow-up

**DOI:** 10.1007/s00701-023-05488-8

**Published:** 2023-01-11

**Authors:** Pavlina Lenga, Gelo Gülec, Awais Akbar Bajwa, Mohammed Issa, Karl Kiening, Andreas W. Unterberg, Basem Ishak

**Affiliations:** Department of Neurosurgery, Heidelberg University Hospital, Im Neuenheimer Feld 400, 69120 Heidelberg, Germany

**Keywords:** Cervical epidural abscess, Octogenarians, Motor deficit, Corpectomy, ACDF

## Abstract

**Purpose:**

Cervical spinal epidural abscess (CSEA) is a rare condition, manifesting as rapid neurological deterioration and leading to early neurological deficits. Its management remains challenging, especially in patients older than 80 years. Therefore, we aimed to compare the clinical course and determine morbidity and mortality rates after anterior cervical discectomy and fusion (ACDF) versus corpectomy in octogenarians with ventrally located CSEA at two levels.

**Methods:**

In this single-center retrospective review, we obtained the following from electronic medical records between September 2005 and December 2021: patient demographics, surgical characteristics, complications, hospital clinical course, and 90-day mortality rate. Comorbidities were assessed using the age-adjusted Charlson comorbidity index (CCI).

**Results:**

Over 16 years, 15 patients underwent ACDF, and 16 patients underwent corpectomy with plate fixation. Between the two groups, patients who underwent corpectomy had a significantly poorer baseline reserve (9.0 ± 2.6 vs. 10.8 ± 2.7; *p* = 0.004) and had a longer hospitalization period (16.4 ± 13.1 vs. 10.0 ± 5.3 days; *p* = 0.004) since corpectomy lasted significantly longer (229.6 ± 74.9 min vs. 123.9 ± 47.5 min; *p* < 0.001). Higher in-hospital and 90-day mortality and readmission rates were observed in the corpectomy group, but the difference was not statistically significant. Both surgeries significantly improved blood infection parameters and neurological status at discharge. Revision surgery due to pseudoarthrosis was required in two patients after corpectomy.

**Conclusions:**

We showed that both ACDF and corpectomy for ventrally located CSEA can be considered as safe treatment strategies for patients aged 80 years and above. However, the surgical approach should be carefully weighed and discussed with the patients and their relatives.

## Introduction

A spinal epidural abscess (SEA) is a rare but devastating illness located between the spinal dura and vertebral periosteum. Specifically, cervical SEA (CSEA) is a rarer clinical condition, accounting for less than 10% of pyogenic infections of the spine [12, 21]. Compared to thoracic or lumbar SEA, CSEA manifests with rapid neurological deterioration, leading to early neurological deficits [12, 20]. Owing to the high risk of septic and neurological complications, urgent treatment is mandatory; hence, the timely use of diagnostic tools is important for preventing such severe events [21].

Confounding factors that might serve as surrogates for the occurrence of CSEA include intravenous (IV) drug abuse, immunodeficiency, increase in comorbidities such as diabetes mellitus type II, a steep increase in spinal surgery, obesity, and an aging population [8, 17, 18].

In nearly all regions of the world, the aging population is growing faster than the total population, thus challenging existing health services [13]. Since older patients, especially octogenarians, are more prone to spinal infections due to their poor baseline reserve, early diagnosis and therapy are paramount to preserving their quality of life. Older patients are at a higher risk of delayed or even missed diagnosis due to compounding comorbidities, which contribute to significantly worse functional outcomes, morbidity, and mortality rates (Amadoru et al., 2017). Little is known about the optimal treatment for such a debilitating cohort, even in the presence of severe neurological deficits. Surgical management with concomitant IV antibiotics might be a key tool for CSEA therapy in younger patients. In the case of octogenarians, due to their unique needs, several variables that affect outcomes related to age should be considered before deciding on a surgical approach.

For instance, ventrally located CSEA requires wide eradication of necrotic tissue followed by anterior cervical discectomy and fusion (ACDF) or even corpectomy when a significant component of osteomyelitis is present, whereas for dorsally located CSEA, combined approaches have been suggested [23]. However, the extent to which these approaches can be performed in a frail cohort and provide clinical benefits remains unclear.

Owing to the lack of robust clinical evidence, we designed the current study to compare and assess the clinical course and to determine morbidity and mortality rates after ACDF and corpectomy exclusively in octogenarians with ventrally located CSEA at a maximum of two levels.

## Methods

Imaging and clinical data were retrospectively collected from our institution’s database between September 2005 and December 2020. This study was approved by the local ethics committee of our institution (no. 880/2021) and was conducted in accordance with the Declaration of Helsinki. The requirement for informed consent was waived owing to the retrospective nature of the study. Patients aged ≥ 80 years with CSEA were enrolled consecutively. The diagnosis was based on magnetic resonance imaging. Spine stability was examined using computed tomography. Exclusion criteria were age < 80 years, concurrent intracranial or cervical pathology, unavailable data, spinal instability, including bony deconstruction resulting in kyphosis or subluxation of the vertebral column, vertebral collapse of > 50% or bone necrosis, and complete loss of disc height. Patient demographics, comorbidities, American Society of Anesthesiologists scores, duration of surgery, number of treated spinal levels, peri- and postoperative complications, hospital length of stay, intensive care unit (ICU) stay, readmission, reoperation, and mortality were obtained from electronic medical records. Comorbidities present before surgery were assessed using the age-adjusted Charlson comorbidity index (CCI) [9, 10]. The CCI was calculated for each patient and classified as no (CCI = 0), minimal (CCI = 1 or 2), moderate (CCI = 3–5), or severe (CCI > 5) comorbidity. The pretreatment neurological condition was assessed using the modified Japanese Orthopaedic Association (mJOA) score for cervical myelopathy [5]. Post-treatment mJOA data were obtained from the last documented clinical encounter. Routine clinical and radiological follow-up examinations were performed before discharge and 3 months after surgery. The final follow-up ranged between 3 and 52 months postoperatively. Standard radiographs in the anteroposterior and lateral views were obtained to evaluate the screw position and fusion rate.

### Surgical procedures

**Fig. 1:**
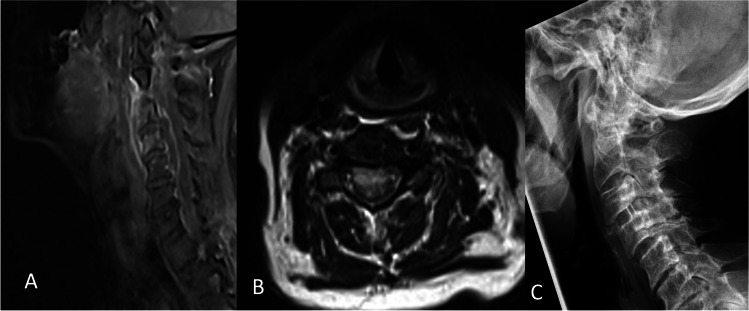
Postcontrast sagittal (**A**) and axial (**B**) T2-weighted magnetic imaging of ventral cervical epidural abscess of C3 and C4 of an 85-year-old male patient presenting with progressive tetraparesis. Lateral radiographic view of ACDF extending from level C3 to C4 with interbody graft seen at C3–C4 (**C**)

**Fig. 2:**
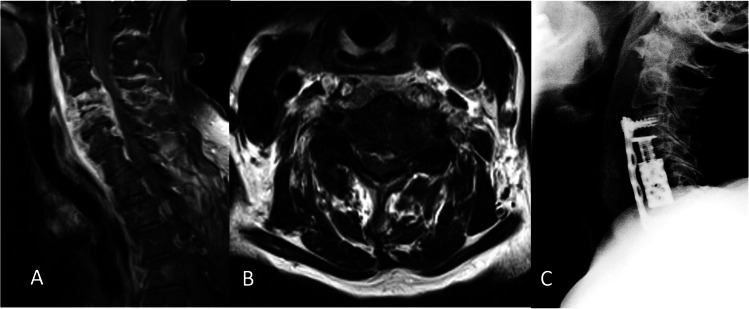
Postcontrast sagittal (**A**) and axial (**B**) T2-weighted magnetic imaging of ventral cervical epidural abscess of C3 and C4 and early endplate destruction of C4 and C5 of an 90-year-old female patient presenting with progressive weakness of low extremities. Lateral radiographic view of corpectomy with placement of a ventral at C4–C5 (**C**)

Patients were allocated to the ACDF or corpectomy group. Decision-making was guided by the presenting neurological status, concomitant underlying pathologies, extent of the pathology, and discretion of an experienced treatment team consisting of neurosurgeons, neuroradiologists, and anesthesiologists. The final decision for ACDF or corpectomy with plate fixation was made according to the surgeons’ preference. Two examples of the surgical procedures are provided in Figs. 1 and 2. In line with our institutional treatment protocols, blood samples or intraoperative cultures were collected before administering IV antibiotics. Thereafter, IV antibiotics were immediately initiated. After identifying the bacterial specimens, the choice of IV antibiotics was adapted to reflect the antibiogram results.

### Statistical analysis

Categorical variables are presented as numbers and percentages. Continuous variables are presented as means ± standard deviations and were verified as normally distributed using the Shapiro–Wilk test. Baseline and surgical characteristics were compared group-wise using independent *t*-tests for continuous variables and chi-squared tests for categorical variables. The Wilcoxon rank test was used to evaluate changes in the C-reactive protein (CRP) level, leukocyte count, and neurological status (mJOA) of each group at discharge. Since these procedures are not commonly applied in patients aged > 80 years and because our sample was relatively small, we could not perform a multivariate analysis to adjust for potential confounders. Statistical significance was set at a *p* value of 0.05.

## Results

### Patient baseline characteristics

Over 16 years, 31 patients aged ≥ 80 years who were diagnosed with CSEA were enrolled in the present study. The mean age was 82.2 ± 1.7 years with a predominance of male patients (*n* = 25/31, 80.6%). Fifteen patients underwent two-level ACDF, and 16 underwent one-level corpectomy with plate fixation. Regarding comorbidities, patients who underwent corpectomy had a significantly poorer baseline reserve than those who underwent ACDF, as measured by the age-adjusted CCI (9.0 ± 2.6 vs. 10.8 ± 2.7; *p* = 0.004). Blood infection parameters were relatively high before surgery in both groups (ACDF: CRP, 156.5 ± 12.7 mg/L; leukocytes, 13.6 ± 5.6 × 10^9^/L vs. corpectomy: CRP, 120.0 ± 22.3 mg/L; leukocytes, 13.1 ± 8.0 × 10^9^/L; *p* > 0.05). Notably, both groups presented with high degrees of disability, as determined by the mJOA. A detailed breakdown of the patient demographics and baseline characteristics is presented in Table 1.

**Table 1:** Baseline patient characteristics

	ACDF *N*^a^ = 15	Corpectomy *N* = 16	*P* value
Age, y (mean, SD^b^)	81.1 (1.3)	82.3 (0.9)	0.066
Sex (*n*^c^, %)			0.318
Male	11 (73.3)	14 (87.5)	
Female	4 (26.7)	2 (12.5)	
BMI^d^, kg/m^2^ (mean, SD)	28.8 (5.4)	26.3 (4.2)	0.347
Comorbidities			
Age-adjusted CCI^e^ score (mean, SD)	10.8 (2.7)	9.0 (2.6)	**0.004**
Arterial hypertension (*n*, %)	12 (80.0)	13 (81.3)	0.930
Myocardial infarction (*n*, %)	11 (73.3)	6 (37.5)	0.916
Coronary heart disease (*n*, %)	11(73.3)	11 (68.8)	0.779
Atrial fibrillation (*n*, %)	4 (26.7)	5 (31.3)	0.779
Heart failure (*n*, %)	5 (33.3)	1 (6.3)	0.056
COPD^f^ (*n*, %)	6 (40.0)	6 (37.5)	0.886
Diabetes mellitus type II (*n*, %)	5 (33.3)	9 (56.3)	0.200
Renal failure (*n*, %)	8 (53.3)	6 (37.5)	0.376
Liver disease (*n*, %)	5 (33.3)	6 (37.5)	0.809
Gastrointestinal ulcer (*n*, %)	4 (26.7)	4 (25.0)	0.916
TIA^g^/stroke (*n*, %)	3 (20.0)	2 (12.5)	0.570
Malignancy (*n*, %)	5 (33.3)	1 (6.3)	0.200
Dementia (*n*, %)	5 (33.3)	2 (12.5)	0.166
Previous spinal surgery (*n*, %)	1 (6.7)	2 (12.5)	0.583
ASA^h^ class (*n*, %)			0.809
II	2 (13.3)	2 (12.5)	
III	11 (73.3)	11 (68.8)	
IV	2 (13.3)	2 (12.5)	
V	0 (0.0)	1 (6.3)	
CRP^i^ level, mg/L (mean, SD)	156.5 (12.7)	120.0 (22.3)	0.697
Leukocytes, count/L (mean, SD)	13.6 (5.6)	13.1 (8.0)	0.423
Preoperative mJOA^j^ score (mean, SD)	7.9 (4.1)	9.8 (3.3)	0.446

Bold *p* value indicates significant difference

^a^Group size

^b^Standard deviation

^c^Number of patients

^d^Body mass index

^e^Charlson comorbidity index

^f^Chronic obstructive pulmonary disease

^g^Transient ischemic attack

^h^American Society of Anesthesiologists

^i^C-reactive protein

^j^Modified Japanese Orthopaedic Association

### Surgical characteristics, clinical scores, and outcomes

As shown in Table 2, corpectomy lasted significantly longer (229.6 ± 74.9 min) than ACDF (123.9 ± 47.5 min; *p* < 0.001). No significant differences were observed in the number of operating levels or ICU stay. Of note, the corpectomy group presented with significantly longer hospitalization (16.4 ± 13.1 vs. 10.0 ± 5.3 days; *p* = 0.004). A trend toward higher in-hospital and 90-day mortality and readmission rates was observed in the corpectomy group, but the differences were not statistically significant. Moreover, comparable improvements in the neurological condition were observed after both surgical procedures, as measured by the mJOA (ACDF: 12.9 ± 3.0 vs. corpectomy: 13.1 ± 4.8; *p* = 0.572). In the second-stage analysis, we examined the outcomes in relation to treatment strategies. After both types of surgery, a significant improvement was observed in blood infection parameters and neurological status at discharge compared with baseline measurements, as displayed in Table 3. The overall mean follow-up period was 26.1 ± 9.8 months. No cases required revision surgery for secondary instability.

**Table 2:** Comparison of surgical characteristics and clinical course between the groups

	ACDF *N*^a^ = 15	Corpectomy *N* = 16	*P* value
Surgical duration, min	123.9 (47.5)	229.6 (74.9)	** < 0.001**
No. of levels decompressed/fused	1.8 (0.2)	2.1 (0.3)	0.495
Hospital stay, days	10.0 (5.3)	16.4 (13.1)	**0.004**
ICU^b^ stay, days	5.9 (6.7)	7.4 (8.6)	0.552
Mortality			
In-hospital (*n*^c^, %)	0 (0.0)	1 (6.3)	0.318
90-day (*n*, %)	1 (6.7)	2 (12.5)	0.083
90-day readmission (*n*, %)	0 (0.0)	1 (6.3)	0.157
Post CRP^d^	98.3 (8.2)	87.2 (10.4)	0.880
Delta CRP	− 61.8 (10.4)	− 34.5 (11.2)	0.780
Post leukocytes	10.2 (3.3)	10.4 (4.9)	0.667
Delta leukocytes	− 3.6 (5.9)	− 2.7 (4.2)	0.780
Post mJOA^e^	12.9 (3.0)	13.1 (4.8)	0.572

Except otherwise indicated, values are presented as the mean (SD) Bold *p* values indicate significant difference

Post, after surgery; Delta, difference between pre- and post-surgical values

^a^Group size

^b^Intensive care unit

^c^Number of patients

^d^C-reactive protein

^e^Modified Japanese Orthopaedic Association

**Table 3:** Comparison of baseline (before surgery) and discharge values

	ACDF Baseline *N*^a^ = 15	ACDF Baseline *N*^a^ = 15	*P* value	Corpectomy Baseline *N* = 16	Corpectomy Discharge *N* = 16	*P* value
CRP^b^	156.5 (12.7)	98.3 (8.2)	**0.054**	120.0 (22.3)	87.2 (10.4)	**0.003**
Leukocytes	13.6 (5.6)	10.2 (3.3)	**0.044**	13.1 (8.0)	10.4 (4.9)	**0.004**
mJOA^c^	7.9 (4.1)	12.9 (3.0)	** < 0.001**	9.8 (3.3)	13.1 (4.8)	** < 0.001**

All data are presented as mean (SD). Bold *p* values indicate significant difference

^a^Group size

^b^C-reactive protein

^c^Modified Japanese Orthopaedic Association

### Complications

The most prevalent complications across both groups were renal failure, heart failure, and pneumonia. However, no significant intergroup differences were observed. Dysphagia was present in three patients undergoing ACDF, although revision surgery was not required. Of note, revision surgery was performed during hospitalization in two patients from the corpectomy group due to pseudoarthrosis with cage dislocation. Posterior decompression and instrumentation were subsequently performed. Details of all the recorded complications are provided in Table 4. *Staphylococcus aureus* was detected—in either blood or intraoperative samples—in approximately half of the patients in the ACDF (60.0%) and corpectomy (50.0%) groups. The next most frequent pathogen in both groups was *Escherichia coli* (ACDF: 13.3% vs. corpectomy group: 18.8%), followed by *Enterococcus faecalis* (ACDF: 13.3% vs. corpectomy: 12.5%) and *Pseudomonas aeruginosa* (ACDF: 6.7% vs. corpectomy: 6.3%), respectively. No pathogens were identified in 6.7% of the ACDF group and 12.4% of the corpectomy group.

**Table 4:** Occurrence of adverse events

	ACDF *N*^a^ = 15	Corpectomy *N* = 16	*P* value
Deep wound infection	1 (6.3)	2 (12.5)	0.328
Acute respiratory failure	0 (0.0)	1 (6.3)	0.325
Acute heart failure	1 (6.7)	4 (25.0)	0.962
Acute renal failure	2 (13.3)	3 (18.8)	0.678
Pneumonia	4 (26.7)	3 (18.8)	0.789
Dysphagia	2 (13.3)	1 (6.3)	0.505
Urinary tract infection	1 (6.7)	1 (6.3)	0.334
Revision surgery	0 (0.0)	2 (12.5)	0.733

All data are presented as number of patients (%). Bold *p* values indicate significant difference

^a^Group size

## Discussion

The cervical spine is an infrequent site of spinal infections, with an incidence ranging between 2 and 19% [21, 24]. Despite the lower incidence, CSEA is associated with higher morbidity and mortality rates [4, 19, 22]. Early surgical decompression and IV antibiotics are the current mainstays of treatment, but optimal treatment for octogenarians remains a subject of debate due to their poor clinical vignette.

### Summary of findings

To the best of our knowledge, this is the first study to systematically describe surgical strategies in octogenarians with ventrally located CSEA at two levels. We evaluated the clinical history, neurological status, surgical characteristics, clinical course of the disease, and morbidity and mortality rates over a 2-year follow-up. Interestingly, we found that patients who underwent ACDF had a poorer baseline reserve than those who underwent corpectomy; there were no significant differences observed between the groups concerning the neurological condition or the levels of infection. It is important to highlight that ACDF was a substantially shorter surgery with a shorter hospitalization stay; however, this did not have any impact on the occurrence of postoperative complications. Notably, a trend toward higher in-hospital and 90-day mortality was observed in the corpectomy group, but this finding was not statistically significant. Irrespective of the treatment, significant improvements in neurological status and decreases in infection parameters were observed after surgery in both groups.

### Review of literature

While surgical procedures for orthopedic, cardiac, or vascular diseases in older patients are becoming widely accepted, surgery for spinal infections, such as CSEA, is viewed with some hesitation for the same patient cohort [14]. In the present study, it is possible that patients with a history of severe underlying pathologies were allocated to ACDF rather than corpectomy because ACDF was associated with a shorter surgical duration and hospitalization. Notwithstanding, a poor baseline history as measured by the age-adjusted CCI did not pose a barrier to early surgery since each patient presented with acute neurological deterioration; hence, surgery was mandatory to preserve or improve patients’ quality of life. Adogwa et al. evaluated 82 patients older than 50 years suffering from SEA, of whom only 12% presented with a cervical location, and reported multiple medical problems, such as diabetes mellitus (32%), malignancy (24%), and end-stage renal problems (9%) [1]. In another study of 30 patients with CSEA with a mean age of 61 years (range 43–88 years), who underwent surgery using a single ventral approach (ACDF, corpectomy) or a combination of approaches, diabetes mellitus, ischemic heart disease, and renal failure were notably prevalent [21]. However, the major limitation in both studies is that the patients were mostly younger, with substantially lower comorbidity profiles compared to our study. Thus, concerns regarding subsequent intra- or perioperative complications were not notable. In a recent study involving our study group, which exclusively focused on octogenarians with thoracic or lumbar SEA, a poor baseline reserve (CCI > 6) was not considered a reason for avoiding surgery since patients presented with acute neurological deterioration [15]. Akin to these findings, we feel that it is imperative to perform surgery for CSEA even in a patient with debilitating conditions to hinder the progression of neurological decline and preserve the quality of life.

Patients with higher comorbidity grades underwent ACDF. It should be noted that a significantly shorter surgical duration and hospitalization stay were observed in the ACDF group. These findings suggest that corpectomy might be associated with higher complication rates due to a longer operative duration and hospital stay. In a recent study on clinical and radiological outcomes in 30 patients with CSEA undergoing surgery, Shousha et al. described 13 cases of ACDF and four cases of corpectomy [21]. Good fusion and acceptable restoration of lordosis were achieved with both surgical techniques, although two cases from the corpectomy group needed revision with additional posterior fixation due to graft dislocation. In line with these findings, Ghorbrial et al., in their series of 40 patients with CSEA, reported four cases of corpectomy involving one or two levels, with good fusion rates. However, one case presented with a high grade of pseudarthrosis at 8 months of follow-up (25.0%), which required posterior stabilization [12]. Likewise, in our study, two patients received additional posterior instrumentation due to dislocation of the pseudoarthrosis after corpectomy, while none of the patients with ACDF required further surgery. Muzii et al. described nine patients with CSEA who underwent one- or two-level ACDF with the concomitant administration of antibiotics. Interestingly, none of the patients underwent revision surgery, and fusion occurred in all cases. However, there are limited data concerning revision rates or infection recurrence. To date, no study has extensively discussed delayed instability that requires revision surgery. It should be emphasized that most studies either omitted reporting such findings due to insufficient follow-up data or the rates were near zero [23]. In the present study, non-surgery-related 90-day readmission was obtained in one case from the corpectomy group. The overall pseudoarthrosis rate after corpectomy was 12.5% and was relatively comparable to rates in previous studies [12, 23]. Similarly, in our study, no revisions were required due to secondary instability over a 2-year follow up. Irrespective of the long-term revision rates, it appears that ACDF provides several benefits and may be considered as a first-line intervention when surgery is considered for older adults.

Shorter surgical time and hospital stay are associated with lower rates of complications and in-hospital infections, thus contributing to lower mortality rates. In our study, we did not find any intergroup differences; however, a trend toward higher complication rates was observed in the corpectomy group. Acute heart and renal failure and pneumonia were the most prevalent complications in both groups, whereas dysphagia was a common complication in the ACDF group. However, the evidence of complications after surgical management for CSEA remains unclear. It is well known that the prevalence of hospital-acquired infections (HAIs) increases linearly with increasing age [6]. Respiratory tract infections are the most common HAI in the geriatric population, followed by urinary tract infections [6]. In particular, octogenarians with multi-morbid diseases and concomitant spinal infections have a very high risk for HAIs [7]. Moreover, acute renal failure is also a common postoperative complication in older patients because of preoperative reduction and multiple comorbidities, as shown in the present study. In the clinical setting, acute renal failure may occur with a postoperative incidence of 20% in older patients and is associated with higher mortality rates [16]. Close monitoring and fluid management are mandatory for such a frail cohort, especially in cases of severe spinal cord infection, which requires antibiotic management, thus hampering normal renal function.

The benefits of early surgery for CSEA with or without neurological deficits remain a contentious issue. In a retrospective analysis of 40 patients with CSEA undergoing surgery, Ghobrial et al. reported improvements in the neurological status of half of the cohort presenting with a neurological deficit at discharge. It should be noted that their cohort consisted mainly of patients with mild neurological deficits (73% presenting with ASIA D or E), which can explain the neurological improvement of only 1 point [12]. No special report has been published concerning the potential amelioration of laboratory findings. The same group, in a subsequent study of 59 patients who had cervical spondylodiscitis with SEA, highlighted the importance of surgery for the improvement of neurological deficits; however, early surgery within the first 24 h after the occurrence of motor weakness was not a significant predictor of a better functional outcome [11]. In contrast, Schimmer et al., in their analysis of 50 patients with CSEA, concluded that early and aggressive intervention for the eradication of CSEA resulted in the complete resolution of neurological deficits in more than 50% of the patients [20]. In the largest retrospective series on CSEA conducted by Alton et al., they advocated that early surgery within less than 24 h after neurological deterioration, along with the administration of IV antibiotics, leads not only to a significant decrease in the infection parameters (CRP, leukocytes) but also to a significant improvement in motor function, thus preserving the quality of life of patients [3]. Medical management alone was related to a high failure rate of 75%, indicating the necessity of a tailored surgical approach [3]. Another systematic review suggested that early surgery might be the key tool for full neurological recovery; nevertheless, potential complications associated with the surgical approach should not be underestimated [23]. Consistent with these findings, we also feel that early surgery in cases of acute neurological deficit might be the optimal treatment even in octogenarians since infection levels were already significantly diminished at discharge and the mean mJOA was approximately 13, which indicated moderate disability, irrespective of the surgical approach.

According to our findings, the mortality rates did not differ between the groups, with in-hospital mortality at 6.3% and 90-day mortality ranging between 6.7 and 12.5%. Of note, a trend toward higher mortality rates was observed in the corpectomy group, a phenomenon that might be attributable to the longer duration of surgery or even the worse baseline of the patients. In a previous study of 50 patients with CSEA undergoing surgery, the mortality rates were similar to those presented here (approximately 5–10%) [21]. In another study of 82 patients older than 50 years, the mortality rate for the surgical cohort was 11.0% [1]. In line with these findings, Shousha et al. also reported a mortality rate of 10% after surgical procedures. Notably, the major distinction between these studies and our findings was that their cohorts consisted of younger patients with lower rates of underlying diseases. Ostensibly, we saw that irrespective of the surgery type (ACDF vs. corpectomy), early surgery might be the best option even in the case of octogenarians since the mortality rates were not notably different from the ones described for younger patients. Considering the significant improvements in infection and neurological condition, physicians should recommend surgery to mitigate disease progression.

### Limitations

The main strength of the current study is that it is the first to examine the outcomes of octogenarians undergoing surgery for two-level CSEA. However, this study has some limitations. First, we examined a relatively small cohort of patients. Nevertheless, since CSEA is scarce and rigorous evidence of its clinical course in octogenarians is still lacking. Therefore, we believe our findings greatly clarify the clinical picture. Second, the minimum follow-up period of 12 months was relatively short. By gathering long-term data, other relevant findings not captured in the current study might have been revealed. Third, as this was a retrospective study, selection bias may have been present.

### Conclusions

Due to a steady increase in average life expectancy, spine surgeons frequently encounter older patients, even octogenarians, requiring surgical therapy for devastating illnesses such as CSEA. Our results show that both ACDF and corpectomy for ventrally located CSEA can be considered safe treatment strategies for patients older than 80 years. A trend toward quicker clinical improvement with lower complication rates was observed in older patients undergoing only ACDF. However, it is imperative to clearly discuss the benefits and potential risks associated with each surgical approach with the patient and their relatives.

## Data Availability

The datasets generated during and/or analyzed during the current study are available from the corresponding author on reasonable request.
